# NF-κB Pathway in Autoinflammatory Diseases: Dysregulation of Protein Modifications by Ubiquitin Defines a New Category of Autoinflammatory Diseases

**DOI:** 10.3389/fimmu.2017.00399

**Published:** 2017-04-19

**Authors:** Ivona Aksentijevich, Qing Zhou

**Affiliations:** ^1^Inflammatory Disease Section, National Human Genome Research Institute, Bethesda, MD, USA

**Keywords:** TNFAIP3/A20, linear ubiquitin chain assembly complex, OTULIN, haploinsufficiency of A20, LUBAC deficiency, otulipenia/otulin-related autoinflammatory syndrome

## Abstract

Autoinflammatory diseases are caused by defects in genes that regulate the innate immunity. Recently, the scope of autoinflammation has been broadened to include diseases that result from dysregulations in protein modifications by the highly conserved ubiquitin (Ub) peptides. Thus far these diseases consist of linear ubiquitin chain assembly complex (LUBAC) and OTULIN deficiencies, and haploinsufficiency of A20. The LUBAC is critical for linear ubiquitination of key signaling molecules in immune response pathways, while deubiquitinase enzymes, OTULIN and TNFAIP3/A20, reverse the effects of ubiquitination by hydrolyzing linear (Met1) and Lys63 (K63) Ub moieties, respectively, from conjugated proteins. Consequently, OTULIN or A20-deficient cells have an excess of Met1 or K63 Ub chains on NEMO, RIPK1, and other target substrates, which lead to constitutive activation of the NF-kB pathway. Mutant cells produce elevated levels of many proinflammatory cytokines and respond to therapy with cytokine inhibitors. Patients with an impairment in LUBAC stability have compromised NF-kB responses in non-immune cells such as fibroblasts, while their monocytes are hyperresponsive to IL-1β. Discoveries of germline mutations in enzymes that regulate protein modifications by Ub define a new category of autoinflammatory diseases caused by upregulations in the NF-kB signaling. The primary aim of this review is to summarize the latest developments in our understanding of the etiology of autoinflammation.

## Introduction

Autoinflammatory diseases are a diverse group of inherited conditions characterized by early-onset systemic inflammation and are accompanied by a range of organ-specific manifestations. The genetic etiology involves abnormalities in molecules such as inflammasomes, cytokine inhibitors, cytokine receptors, enzymes, and proteasome complex. The excessive secretion of proinflammatory cytokines can lead to chronic morbidity and may be life-threatening. Therapies with cytokine inhibitors are efficacious in most patients; however, considering the high cost of biologics there is a need to develop more affordable treatment options for these lifelong conditions. The discovery of germline mutations linked to autoinflammation offer an opportunity to search for new therapeutic targets.

## Ubiquitin (Ub) Pathway

Posttranslational protein modification by ubiquitination (also known as ubiquitylation) is critical for the regulation of many biological processes including DNA repair, endocytosis, transcription, protein degradation, and preservation of cellular homeostasis. Ubiquitination involves the attachment of evolutionarily conserved 76-aa Ub molecules to target proteins in the form of a monomer or polymers (Ub chains). The type of conjugation determines the fate of the modified protein by directing protein localization and regulating protein interactions, activity, and degradation (1).

The ubiquitination process is initiated by the attachment of a single Ub molecule to a target protein through a three-step enzymatic pathway that includes Ub-activating enzymes (E1), Ub-conjugating enzymes (E2), and Ub-ligating enzymes (E3) (2). Ub chains are generated by the sequential addition of Ub monomers through one of seven lysine residues that serve as a linker, thus there are seven types of Ub Lys-linkages. Ub can be also conjugated through an N-terminal methionine residue (Met1 linkage; linear linkage). Lys11-, Lys48-, Lys63-, and Met1-linked chains are the best known and most studied. Significance of four other Ub chains Lys6-, Lys27-, Lys29-, and Lys33- is poorly understood. In addition, there is increasing evidence that more than one linkage type can exist on modified proteins either in the form of mixed (hybrid) Ub chains or branched Ub chains, and this may provide a new layer of complexity to the Ub-mediated modifications (2, 3). Ub proteins are also subject to modifications by acetylation, phosphorylation, and ubiquitin-like (UBL) molecules (SUMO or NEDD8) (4). Ub-conjugated proteins are recognized by Ub sensors, Ub-binding proteins (Ub receptors) that can translate each linkage type of Ub modifications into specific functional outcomes. For example, proteins conjugated with Lys (K11) or Lys48 (K48) Ub chains are targeted for proteosomal degradation *via* the ubiquitin–proteasome system (UPS) (5). In addition, Lys11 Ub chains have a role in cell cycle control and may have other functions in the context of mixed Ub chains (6). Lys63 (K63) Ub chains are involved in cell signaling and are essential for DNA damage response (7). Linear (Met1) Ub chains regulate a wide range of immune signaling pathways (8–10). Ubiquitination is a highly dynamic and reversible process whereby Ub chains are removed from modified substrates by a class of enzymes known as deubiqutylases or deubiquitinases (DUBs) (11). There are close to 100 proteases that have DUB activity with different degrees of specificity for Ub linkages. Several DUBs, including A20, OTULIN, CYLD, and Cezanne, function as negative regulators of NF-kB signaling (12).

Alterations in various components of the Ub–proteasome machinery have been linked to many human conditions including immune diseases. Recently, deregulations in the UPS were reported in patients with autoinflammatory disorders including chronic atypical neutrophilic dermatosis with lipodystrophy and elevated temperature (13), linear ubiquitin chain assembly complex (LUBAC) deficiency (14, 15), haploinsufficiency of A20 (HA20) (16), and otulipenia/otulin-related autoinflammatory syndrome (ORAS) (17, 18). This review will primarily focus on two diseases caused by malfunction in DUB enzymes, TNFAIP3/A20 and OTULIN, which are known to hydrolyze Lys63- and Met1-linked Ub chains, respectively. In both conditions, HA20 and otulipenia/ORAS, a defect in DUB activity results in excessive ubiquitination and increased activity of key signaling molecules in the canonical NF-kB pathway. LUBAC-associated diseases will be briefly discussed in the context of LUBAC–OTULIN interactions.

## Linear Ub Chains in Immune Signaling

Linear ubiquitin chain assembly complex (LUBAC)-mediated Met1 ubiquitination has emerged pivotal for regulation of innate and adaptive immune responses and regulation of cell death (9, 19). The E3 ligase complex, LUBAC, has been shown to maintain the stability of the TNF receptor 1 (TNFR1), TLRs, IL-1R, CD40, RLR, and inflammasome receptor signaling complexes (RSCs). Upon stimulation with proinflammatory signals, LUBAC is recruited to attach linear Ub chains to target substrates such as IKK (NEMO), RIPK1, RIPK2, IRAKs, MyD88, and ASC (8, 20, 21). Attachment of linear Ub chains is critical for the assembly of RSCs. LUBAC depletion leads to attenuation of NF-kB and the mitogen-activated protein kinases (MAPK)-mediated signaling and increases cell death.

**Figure 1:**
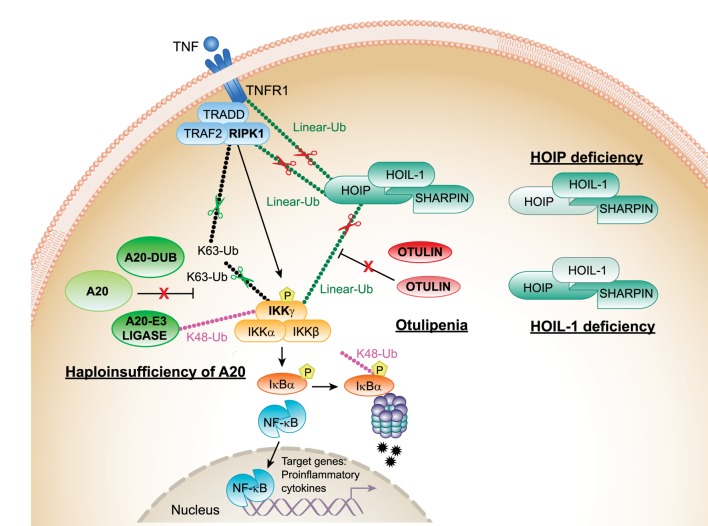
**Proposed mechanisms of pathogenesis in haploinsufficiency of A20 (HA20), otulipenia/otulin-related autoinflammatory syndrome (ORAS), and linear ubiquitin chain assembly complex (LUBAC) deficiencies**. The canonical NF-κB pathway is regulated both by K63 (Lys63)-linked and linear (Met1)-linked ubiquitin (Ub) chains. RIPK1 is the central adaptor for assembly of the TNFR1 receptor signaling complex and is a predominant target for ubiquitination by K63 and linear Ub chains. Polyubiquitylated RIPK1 mediates recruitment of IKK complex that is also target for ubiquitination. The activated IKK complex phosphorylates inhibitor of kappa B (IκBα) and targets IκBα for proteasome-mediated degradation. Linear Ub chains are added to RIPK1 and IKKγ by LUBAC. A20 and OTULIN negatively regulate the NF-κB pathway by cleaving K63 and linear Ub chains from target molecules, RIPK1 and IKKγ. In addition, A20 through its E3 ligase activity adds K48 Ub chains to IKKγ (and TRAF6, not shown in the figure) targeting them for proteasomal degradation. Decreased expression of A20 in patients with HA20 or OTULIN in patients with otulipenia/ORAS will lead to activation of the NF-κB pathway, increased expression of proinflammatory transcripts in immune cells, and systemic inflammation. Decreased expression of LUBAC complex subunits, in patients who carry mutations in HOIP or HOIL-1, results in inhibition of the NF-κB pathway in fibroblasts and B-cells causing immunodeficiency. However, their monocytes hyperproduce proinflammatory cytokines. TNFR1, TNF receptor 1; TRADD, TNFR1-associated death domain protein; RIPK1, the death domain-containing protein kinase receptor-interacting protein1; IKKγ/NEMO, inhibitor of nuclear factor kappa B kinase subunit gamma.

Linear ubiquitin chain assembly complex consists of HOIP (HOIL-1 interacting protein; *RNF31*) and two accessory proteins: HOIL-1 (heme-oxidized IRP2 ubiquitin ligase 1; *RBCK1*) and SHARPIN (SHANK-interacting protein like 1; *SIPL1*) (Figure 1). The catalytic subunit HOIP is auto-inhibited until the complex is fully assembled. Depletion of any subunits greatly reduces the stability of LUBAC in cells (14, 15). As the immune responses must be tightly regulated to avoid chronic inflammation, LUBAC activity is counter-regulated by the specific DUB OTULIN (also known as *gumby*; *FAM105B*).

OTULIN is a highly conserved protease with a specific activity to hydrolize linear (Met1)-linked Ub chains (22) (Figure 1). OTULIN interacts with the N-terminal PUB domain of HOIP *via* an evolutionarily conserved PUB-interacting motif (23). Loss of HOIP–OTULIN interaction reduces OTULIN capacity to restrict LUBAC-induced NF-kB activation (24). A recent study showed that the activity of LUBAC is also negatively regulated by its interaction with tumor necrosis factor receptor-associated factor 1 (TRAF1). TRAF1 directly interacts with LUBAC to interfere with the activation of IKK/NEMO. Reduced expression of TRAF1 could explain the association of susceptibility alleles in *TRAF1* with rheumatoid arthritis (RA) and other autoimmune diseases (25).

The importance of the linear ubiquitination in regulation of inflammatory pathways has been demonstrated in murine models. Genetic ablation of the catalytic HOIP subunit results in embryonic lethality at day 10.5 due to TNF-mediated endothelial cell death and vascular abnormalities (26). Mice deficient for non-catalytic subunits have variable degrees of inflammation manifesting with chronic proliferative dermatitis in the case of SHARPIN-deficient mice (*cpdm*) (27, 28) or with no overt inflammation in mice lacking HOIL-1 (21). Skin inflammation in Sharpin-KO mice is largely dependent on the TNFR1-induced apoptosis (19, 29, 30). LUBAC activity is also important for proper B and T cell development, activation, and maintenance of adaptive immune responses (31, 32). Genetic loss of *Otulin* (*gumby*/*gumby*) causes embryonic lethality (E12.5–E14) due to compromised angiogenesis and defects in neuronal development (33). Tamoxifen-induced *Otulin* deficiency in immune cells results in an acute severe multiorgan inflammatory phenotype (18). Targeted ablation of *Otulin* in myeloid cells leads to chronic inflammation with features of autoimmunity, while *Otulin* deficiency in adaptive immune cells does not produce overt phenotype (18). Together, these data show critical and cell-specific function of OTULIN in the maintenance of immune homeostasis.

## Lubac Deficiencies

**Figure 2:**
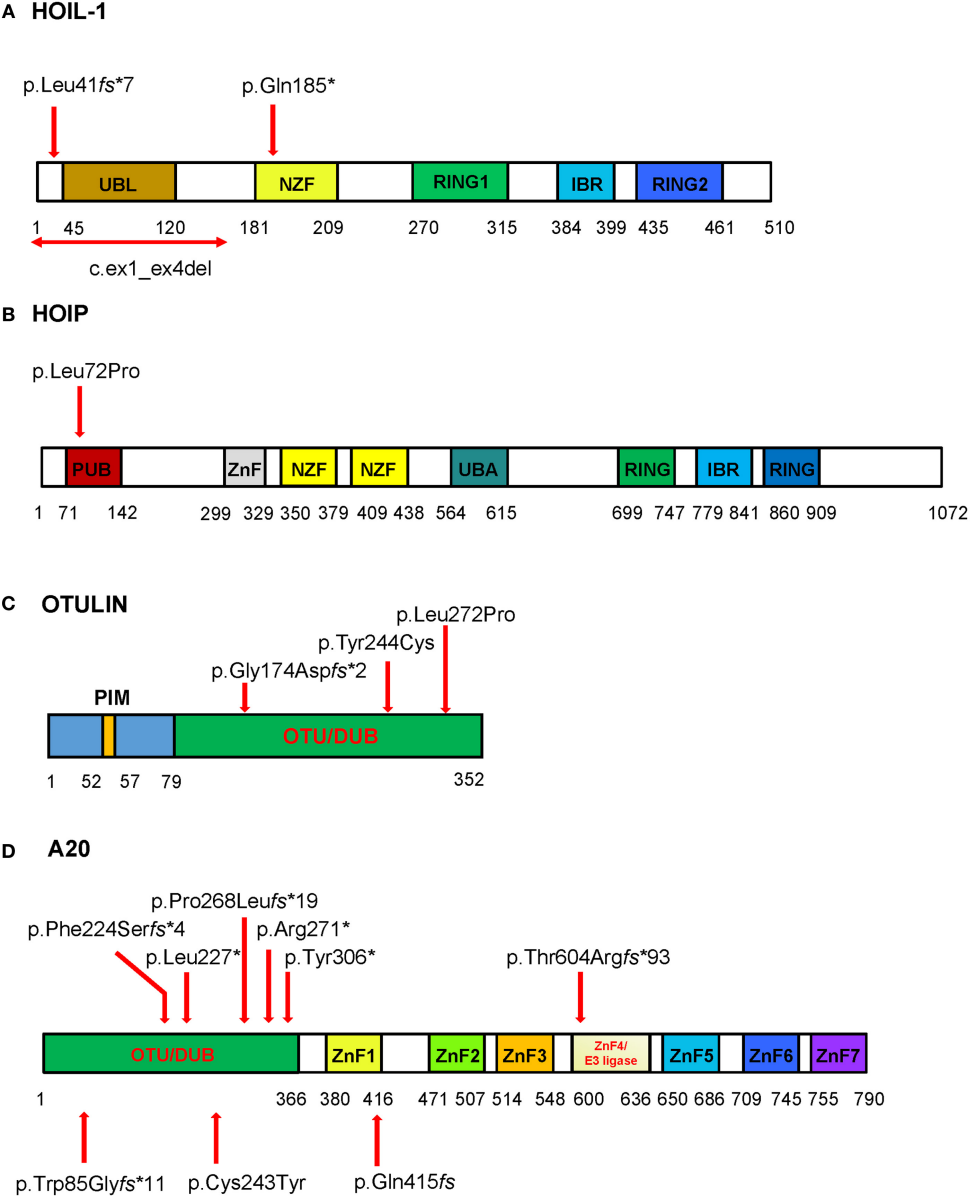
**Schematic of the domains and locations of mutations in respective proteins HOIL-1, HOIP, OTULIN, and TNFAIP3/A20**. The domains identified are depicted as boxes. The locations of the mutations are indicated with red arrows. **(A)** HOIL-1 contains the ubiquitin-like (UBL), NPL4 zinc-fingers (NZF) domain, really interesting new gene (RING) domain, and in-between RING (IBR) domain. The mutations are located in UBL and NZF domains. The UBL domain is required for linear ubiquitin chain assembly complex (LUBAC) formation and linear ubiquitination. **(B)** HOIP has PNGase/UBA or UBX (PUB), zinc finger (ZnF), NZF, ubiquitin-associated (UBA), RING domain, and IBR domain. The homozygous disease-associated mutation is located in the PUB domain that is critical for interaction with OTULIN and stability of LUBAC. **(C)** OTULIN consists of N-terminal LUBAC-binding PUB-interacting motif (PIM) and C-terminal ovarian tumor (OTU) domain that mediates deubiquitinase (DUB) activity of OTULIN (79–352aa). All three mutations are located in the OTU domain. **(D)** The DUB activity of A20 is mediated by the OTU domain, and the ZnF domains mediate A20 ubiquitin (Ub) E3 ligase activity, binding to Lys63-linked Ub chains and dimerization. Mutations reported by Zhou at al. are shown on the top of the diagram, while three mutations reported in Japanese patients are shown bellow the diagram. To stay consistent with the Human Genome Variation Society nomenclature the reported p.Gln415fs (35) should be described as p.Lys417Serfs*4. The four nucleotide deletion in the repeat sequence (reported as c.1245_1248del) should be assigned as the most 3′ position in the repeat, i.e., c.1249_1252del; therefore, the proposed nomenclature for the mutation is p.Lys417Serfs*4.

Patients with defects in the LUBAC components develop immunodeficiency, autoinflammation, muscular amylopectinosis, and die in early childhood (Tables 1 and 2). HOIL-1 and HOIP deficiencies are recessively inherited diseases caused by mutations that either create truncating proteins or affect a highly conserved PUB domain of HOIP (14, 15) (Figure 2). Pathogenic mutations in one subunit destabilize the expression of the entire LUBAC complex. As LUBAC is important for activation of immune signaling, stimulated patient fibroblasts and B cells fail to upregulate NF-kB activity, which manifests in recurrent bacterial infections. In contrast to immunodeficient fibroblasts, peripheral blood mononuclear cells (PBMCs) of HOIP and HOIL-1-deficient patients are highly responsive to IL-1 stimulation and produce high levels of proinflammatory cytokines IL-6 and MIP-1α. One patient was noted to have severe T cell lymphopenia and impaired antibody production. Muscular amylopectinosis/myopathy appear to be unrelated to a defect of linear ubiquitination in immune cells and its mechanism remains to be investigated. Abnormalities in the lymphatic system have been observed in the HOIP-deficient patient and HOIP-deficient mice, which suggests that HOIP, independently of other LUBAC components, may have a function in the regulation of angiogenesis. LUBAC appears to be important for toll-like receptor 3 (TLR3)-mediated innate immune responses to influenza A virus infection, by enabling TLR3-mediated activation of NF-kB, MAPK, and IRF3 (34).

**Table 1:** **Comparison of genetics and mechanisms of disease in otulipenia, haploinsufficiency of A20 (HA20), and linear ubiquitin chain assembly complex (LUBAC) deficiencies**.

		Otulipenia/ORAS	HA20
Gene	Gene name	*OTULIN* (*FAM105B*)	*TNFAIP3*
	Exons	7 exons	9 exons
	Chromosome	Chr.5	Chr.6

Protein	Protein name	OTULIN	A20
	Protein length	352aa	790aa
	Protein domains	PUB-interacting motif domain, ovarian tumor (OTU) domain	OTU domain, 7 ZnF domains
	Protein function	Met1 linear deubiquitinase (DUB)	K63 DUB
	Involved pathway	NF-κB	NF-κB, NLRP3
	Substrate molecules	NEMO, TNF receptor 1 (TNFR1), RIPK1, ASC	NEMO, RIPK1, TRAF6, pro IL-1β

Genetics	Inheritance	Recessive	Dominant
	Type of mutations	Loss-of-function mutations (missense, INDELS)	Loss-of-function mutations (stop gain mutation, missense, INDELS)
	Frequency of mutant alleles	Rare or novel	Novel
	Location of the mutations	OTU domain (linear DUB activity)	OTU domain (k63 DUB activity) or ZnF4 domain
	Number of mutations	Biallelic (compound heterozygous or homozygous)	Heterozygous

Mechanisms	Mechanism	Loss-of-function (reduced protein expression)	Haploinsufficiency (50% decrease in protein expression)
	Protein Interactions	Instability of LUBAC	Decreased association with TNFR1, TRAF2, and RIPK1
	Effect of mutant proteins	Impaired linear deubiquitination of NEMO, TNFR1, RIPK1, ASC	Impaired K63 deubiquitination of NEMO, RIPK1, and TRAF6
	Involved pathway	Increased signaling in NF-κB and mitogen-activated protein kinases (MAPK) pathways	Increased activity of NF-κB, MAPKs, and NLRP3
	Cytokines	IL-1β, TNF, IL-6, IL-12, IL-18, IFNγ	IL-1β, TNF, IL-6, IL-9, IL-17, IL-18, IFNγ

		**HOIL-1 deficiency**	**HOIP deficiency**

Gene	Gene name	*RBCK1*	*RNF31*
	Exons	12 exons	21 exons
	Chromosome	Chr.20	Chr.14

Protein	Protein name	HOIL-1	HOIP
	Protein length	510aa	1,072aa
	Protein domains	Ubiquitin-like (UBL), novel zinc finger (NZF), RING1, IBR, RING2	PUB, ZnF, NZF1, NZF2, UBA, RING1, IBR, RING2, LDD
	Protein function	Subunit of the LUBAC	Catalytic subunit of the LUBAC
	Involved pathway	NF-κB signaling pathway	NF-κB signaling pathway

Mutations	Type of mutations	Loss-of-function mutations (stop gain mutation, INDELS)	Loss-of-function mutations (missense)
	Frequency of mutant alleles	Rare or novel	Novel
	Location of the mutations	UBL domain (interacts with HOIP UBA domain), NZF domain (ubiquitin binding)	PUB domain (interacts with OTULIN)
	Number of mutations	Biallelic (compound heterozygous or homozygous)	Biallelic (homozygous)

Mechanisms	Inheritance	Recessive	Recessive
	Mechanism	Loss-of-function (decreased protein expression, instability of LUBAC, impaired linear ubiquitination)	Loss-of-function (decreased protein expression, instability of LUBAC, impaired linear ubiquitination)
	Effect of mutant proteins	Defect in linear ubiquitination NEMO, RIPK1, IRAK-1	Defect in linear ubiquitination
	Involved pathway	Impaired NF-κB activation in fibroblasts, increased NF-κB activity in monocytes	Impaired NF-κB activation in fibroblasts and CD40L stimulated B cells, increased NF-κB activity in monocytes
	Cytokines	Impaired expression of IL-6 in fibroblasts upon IL-1β and TNF stimulation; hyperproduction of IL-6 upon in IL-1β stimulated monocytes; high serum IL-1, IL-6	Hyperproduction of IL-6 in IL-1β stimulated monocytes

**Table 2:** **Clinical manifestations in patients with otulipenia, haploinsufficiency of A20 (HA20), and linear ubiquitin chain assembly complex deficiencies**.

Clinical manifestations	Otulipenia (17)		HA20 (16, 35–37)					HOIL-1 deficiency (14)		HOIP deficiency (15)
	Yes or no	Patients (*n* = 3)	Yes or no	Patients (16) (*n* = 11)	Patients (37) (*n* = 6)	Patients (36) (*n* = 3)	Patients (35) (*n* = 1)	Yes or no	Patients (*n* = 3)	Yes or no (*n* = 1)
Early age onset	Yes (1–4.5 months)	3/3	Yes (7 months–16 years)	11/11	6/6	3/3	1/1	Yes	3/3	Yes

Fevers	Yes (fever lasting 2–3 weeks)	3/3	Yes	2/11	3/6	1/3	1/1	Yes	3/3	Yes

Skin rash	Yes (erythematous with skin nodules, pustular rash)	3/3	Yes (erythematous papules, folliculitis, skin abscesses)	4/11	6/6	0/3	1/1	Yes (eczema; erythroderma, desquamative dermatitis)	1/3; 1/3	No

CNS	No	1/3	Yes (CNS vasculitis, chorea, migraine)	2/11	/	/	0/1	No	0/3	No

GI	Yes (abdominal pain, diarrhea)	1/3	Yes (colitis)	4/11	1/6	1/3	0/1	Yes (abdominal pain, diarrhea, vomiting, blood and mucus in the stools)	3/3	Yes (recurrent episodes of fatty diarrhea, intestinal lymphangiectasia)

Arthritis	Yes (arthralgias, myalgias)	3/3	Yes (arthralgia, polyarthritis)	6/11	/	2/3	0/1	No	0/3	Yes
Arthralgias										
Myalgias										

Elevated CRP, ESR	Yes	3/3	Yes	6/6	1/1	/	1/1	Yes	3/3	Yes

Immunodeficiency	No obvious primary immunodeficiency	2/3	Yes (IgG2 and 4 subclass deficiency, low anti-polysaccharide antibodies lymphopenia)	2/11	/	/	1/1	Yes (recurrent bacterial infections, memory B-cell deficiency, and hyper-IgA syndrome)	3/3	Yes (recurrent viral and bacterial infections, lymphopenia, antibody deficiency, hypogammaglobulinemia)

Oral ulcers	No	0/3	Yes	11/11	6/6	2/3	0/1	Yes	1/3	Yes

Genital ulcers	No	0/3	Yes	10/11	6/6	1/3	0/1	No	0/3	No

Ophtho	No	0/3	Yes (uveitis, retinal vasculitis)	3/11	/	1/3	/	No	0/3	No

Pathergy	No	0/3	Yes	3/11	/	/	/	No	0/3	No

Autoantibodies	No	0/3	Yes (RNP, ANA, lupus anticoagulant)	5/11	/	/	1/1	No	0/3	No

Panniculitis	Yes	3/3	No	0/11	0/6	0/3	0/1	No	0/3	No

Failure to thrive	Yes	3/3	No	0/11	0/6	0/3	0/1	Yes	3/3	Yes

Lipodystrophy	Yes	3/3	No	0/11	0/6	0/3	0/1	No	0/3	No

Lymphadenopathy	Yes	2/3	Yes	0/11	0/6	0/3	1/1	Yes	2/3	Yes

Systemic lymphangiectasia	No	0/3	No	0/11	0/6	0/3	0/1	No	0/3	Yes

Weakness in lower extremities	No	0/3	No	0/11	0/6	0/3	0/1	Yes	2/3	Yes

Cardiomyopathy	No	0/3	No	0/11	0/6	0/3	0/1	Yes	3/3	Yes

Respiratory distress	No	0/3	No	0/11	0/6	0/3	0/1	Yes	1/3	Yes
Amylopectinosis	No	0/3	No	0/11	0/6	0/3	0/1	Yes	3/3	Yes
Systemic edema	No	0/3	No	0/11	0/6	0/3	0/1	No	0/3	Yes
Effective treatment	anti-TNF, anti-IL-1	2/3; 1/3	anti-IL-1, anti-TNF; colchicine	1/11; 4/11; 2/11	0/6; 0/6; 1/6	0/6; 0/6; 0/6	0/1; 0/1; 0/1	anti-TNF, steroids; BMT	2/3; 1/3	Naproxen, antibiotic prophylaxis, IVIG

*/, not reported*.

*Source of data: Ref. (14–18, 35–37)*.

In summary, the identification of patients with LUBAC deficiencies showed the critical role of LUBAC in regulation of immune responses.

## Otulipenia/ORAS

**Figure 3:**
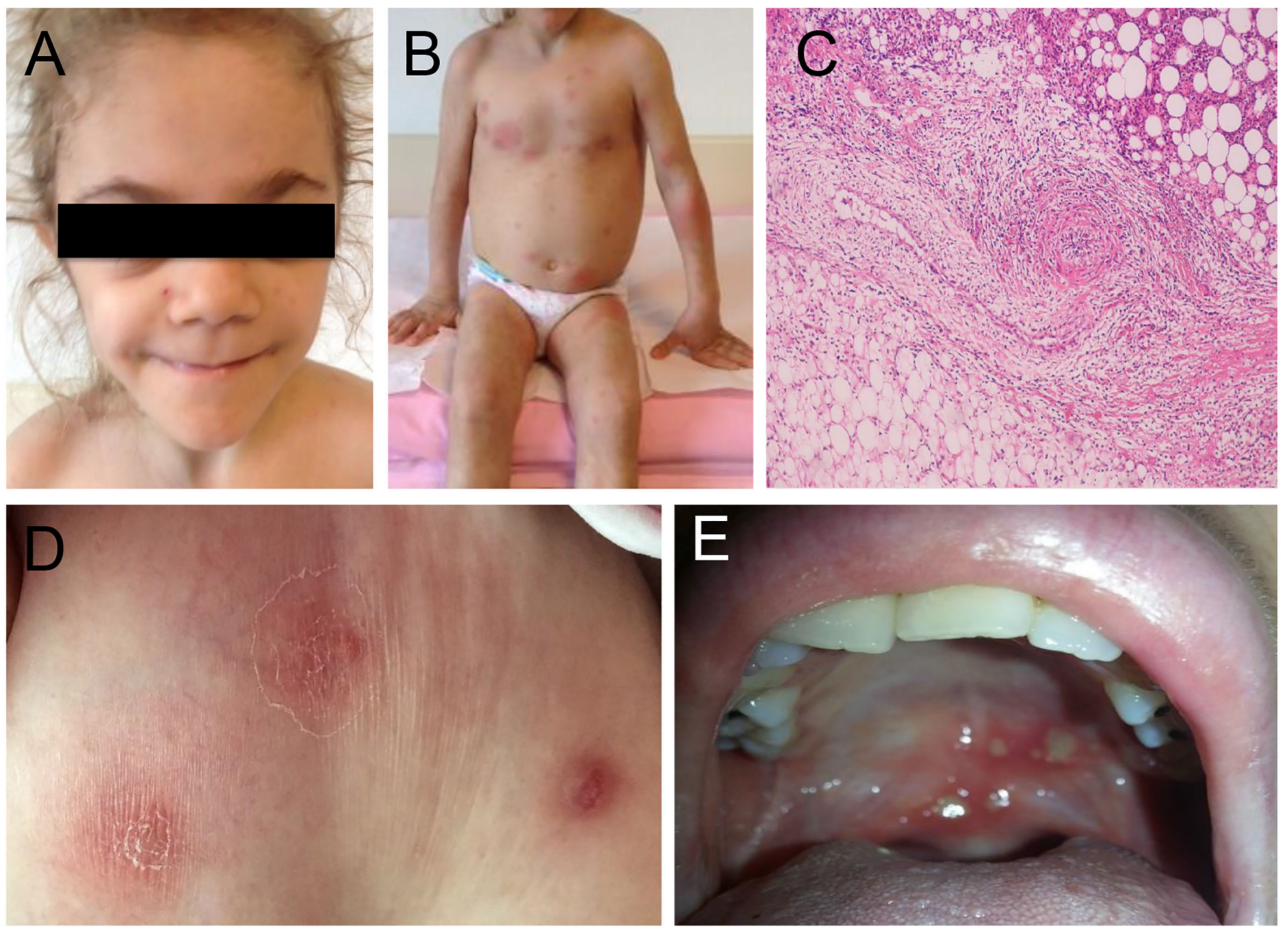
**Clinical manifestations of the patients with haploinsufficiency of A20 (HA20) and otulipenia/otulin-related autoinflammatory syndrome**. **(A)** Prominent fat loss (lipodystrophy) and **(B)** erythematous skin lesions and subcutaneous nodules in a patient with otulipenia. **(C)** Skin biopsy showed dense inflammatory infiltrate throughout the subcutaneous lobules (right upper corner of the image) and subcutaneous lobular atrophy or lipodystrophy (similar findings have been reported in chronic atypical neutrophilic dermatosis with lipodystrophy and elevated temperature patients) on the left lower corner of the image. The middle part of the image showed vasculitis affecting a medium-sized artery, characterized by dense intramural and perivascular inflammation with endothelial proliferation and vascular occlusion. Adjacent the affected artery is a medium-sized vein (left lower) showing mild inflammation of the vessel wall. **(D)** Dermal abscesses in a patient with HA20. **(E)** Recurrent aphthous (oral) ulcers in a patient with HA20.

Recessively inherited loss-of-function mutations in the linear (Met1)-specific DUB OTULIN have been linked to the early-onset severe inflammatory disease, named otulipenia/ORAS (Table 1) (17, 18). Patients present with prolonged recurrent fevers, joint swelling, GI inflammation/diarrhea, and failure to thrive (Table 2; Figure 3). The cutaneous manifestations include painful erythematous rash with skin nodules, lipodystrophy, and episodes of pustular rash in one patient (Figure 3B). Skin biopsy showed evidence for neutrophilic dermatitis, mixed type panniculitis, and vasculitis of small and medium-sized blood vessels (Figure 3C) (17). In contrast to patients with the LUBAC deficiency, OTULIN-deficient patients have no obvious immunodeficiency, although some of them suffered from iatrogenic infections induced by immunosuppressive therapies. Initial analyses showed adequate specific antibody responses to vaccines, adequate T and B cell proliferative responses, normal levels of immunoglobulins, and normal T, B, and NK cell counts.

The four patients identified carry novel homozygous mutations in the *FAM105B* gene that encodes OTULIN. Heterozygote carriers are asymptomatic, which suggests that reduced protein expression of OTULIN may not be critical for maintenance of immune homeostasis. OTULIN is a 352-residue protein that consists of N-terminal LUBAC-binding domain and C-terminal ovarian tumor (OTU) domain. Disease-associated mutations affect the OTU domain and binding of OTULIN to linear Ub chains (Figure 2).

OTULIN functions as a negative regulator of the canonical NF-kB pathway and as such is essential for resolving inflammation (Table 1; Figure 1). Mutant OTULIN proteins cannot restrict the accumulation of Met1 Ub chains on target substrates IKK/NEMO, RIPK1, and ASC. Patients’ mononuclear leukocytes and fibroblasts have a strong inflammatory signature as evidenced by increased degradation of IkBα and increased phosphorylation of IKKα/IKKβ and IkBα, the hallmarks of the activated NF-kB pathway. Mutant cells overproduce many proinflammatory cytokines, including cytokines associated with activation of adaptive immune cells, and therapy with TNF inhibitors is very effective in controlling disease activity. Tamoxifen-induced *Otulin* ablation in murine immune cells (CreERT2-*Otulin*_*LacZ*/flox_ chimeras) resembles the phenotype described in patients with otulipenia/ORAS including responsiveness to therapy with TNF inhibitors (18).

In summary, combined data from human and murine studies suggest that deficiency of the linear DUB, OTULIN, leads to amplification of the Met1 Ub regulated signaling in the canonical NF-kB pathway, most notably in myeloid cells.

## Lys63-Linked Ub Chains in Immune Signaling

Lys(K)63-Ub modification was first described as a mechanism for the activation of the canonical NF-κB pathway (38). K63-linked Ub chains are generated following cell stimulation with inflammatory cytokines, and they function as a scaffold for the formation of receptor signaling complexes. Molecules such as NEMO/IKK, TRAF6, and RIPK1 are ubiquitinated both by linear and K63 Ub chains, which suggest a substantial regulatory redundancy in immune signaling (39–42). TNFAIP3/A20 restricts inflammatory responses *via* its dual yet synergistic functions: its deubiquitinase activity by hydrolyzing K63 linkages and its E3 ligase activity by conjugating substrates with K48 Ub chains to target them for proteasomal degradation (43, 44) (Figure 1). A20 is also subject to regulations as it undergoes posttranslational phosphorylation (45) and is cleaved by mucosa-associated lymphoid tissue lymphoma translocation protein 1 (46). In tumor cells, A20 acts as a tumor-suppressor gene and is frequently inactivated by somatic mutations and deletions in diffuse large B-cell and Hodgkin lymphomas (47, 48).

The *TNFAIP3* gene is highly conserved and intolerant to genetic variations, in particular to loss-of-function mutations. Common low-penetrance mostly non-coding variants in *TNFAIP3* have been associated with many autoimmune diseases including systemic lupus erythematosus (SLE) (49–51), RA (52), psoriasis (53), type 1 diabetes (54), celiac disease (55), coronary artery disease (56), inflammatory bowel disease (57), and more recently with protection to allergy and asthma (58, 59). Given the potent anti-inflammatory function of A20, these susceptibility alleles are predicted to decrease A20 expression and function, although that has been experimentally demonstrated only for a single non-coding variant associated with SLE. The dinucleotide functional variant downstream of *TNFAIP3*, TT > A, likely alters the binding of transcription factors in response to proinflammatory signals (51, 60).

Genetic ablation of A20 leads to spontaneous inflammation in mice with a range of cell-specific phenotypes. A20-deficient (A20^−/−^) mice exhibit multiorgan inflammation, cachexia, and early lethality (61). Although A20 was initially described as required for termination of TNF-induced signals, the excessive inflammation observed in double-deficient mice, A20-TNF or A20-TNFR1, suggested that A20 might be critical for the regulation of TNF-independent signals including the termination of TLR-induced activity of NF-kB (62). Cell-specific deletions of A20 resemble human autoimmune diseases, from a mild autoimmune phenotype in *Tnfaip3 Cd19* (B-cell) KO mice to severe spontaneous inflammation in mice with A20-deficient dendritic cells. Loss of A20 in macrophages mimics human RA, although the phenotype appears to be TNF-independent. Deficiency of A20 in keratinocytes leads to hyperkeratosis, while loss of A20 in intestinal epithelial cells causes DSS-induced TNF-dependent colitis (63). Aging heterozygous mice (A20^+/−^) develop spontaneous autoantibodies. In summary, multiple murine models with cell-specific ablation of A20 demonstrate closer approximation of human diseases than the complete knock-out mice.

This past year, Zhou et al. reported 11 patients from 6 families with a new dominantly inherited autoinflammatory disease, termed haploinsufficiency of A20, characterized by childhood-onset episodic fevers, arthralgia/arthritis, oral and/or genital ulcers, skin pathergy, GI, and ocular inflammation (16) (Table 2; Figures 3D,E). These symptoms resemble Behcet’s disease (BD). One patient was initially diagnosed with SLE and presented with CNS vasculitis and idiopathic thrombocytopenic purpura. Subsequently, two families of Japanese ancestry diagnosed with entero-BD and one Japanese patient diagnosed with autoimmune lymphoproliferative syndrome (ALPS) were reported to carry novel LOF mutations in the gene (Figure 2) (35–37). A patient with ALPS presented with fevers, bilateral cervical lymphadenopathy, extensive skin rash, and massive hepatosplenomegaly. The patient’s immunophenotyping revealed an increased percentage of DNT cells and a decreased number of IgM memory B cells, which is characteristic of ALPS. However, unlike in ALPS the patient’s central memory (TCM), naïve, TEMRA, and effector memory (TEM) subpopulations of CD^3+^CD^8+^ cells were normal (35).

TNFAIP3/A20 is a 790-residue protein that consists of an amino-terminal OTU followed by seven zinc finger (ZnF) domains. HA20-associated mutations create truncated mutant proteins, and most of them are located in the OTU domain (Figure 2). In addition to its OTU domain-mediated DUB activity, A20 can downregulate IKK activation by blocking IKK phosphorylation (64). Two pathogenic mutations have been identified in the ZnF domains 1 and 4. The ZnF4 domain is essential for A20 E3 ligase activity and dimerization (65).

Similar to patients with otulipenia/ORAS, mutant A20 cells have enhanced NF-kB activity as demonstrated by increased phosphorylation of IKKα/β and increased degradation of IkBα. Stimulated patient PBMCs and fibroblasts failed to hydrolyze K63 Ub chains from NEMO/IKK, RIPK1, and TNFR1 (16). Accumulation of K63 Ub proteins on these molecules triggers activity of the NF-kB and the MAPK pathways (Table 1; Figure 1). In murine models, A20/Tnfaip3 was shown to downregulate the activity of NLRP3 inflammasome (40, 66). Zhou et al. demonstrated constitutive NLRP3 activity in PBMCs of HA20 patients. Stimulated PBMCs and serum samples of HA20 patients have highly elevated levels of many proinflammatory cytokines produced by myeloid cells (IL-1, TNF, IL-6, IL-18, and IP-10) and T cells (IL-9, IL-17, and IFNγ). Therapies with cytokine inhibitors, anti-TNF, or anti-IL-1, have been very efficient in suppressing systemic inflammation.

Taken together, human genetic studies and murine models of A20 deficiency provide strong evidence that the reduced expression of A20 is associated with a range of inflammatory phenotypes.

## Conclusion

Maintenance of immune homeostasis is a highly balanced act that requires coordinated action of many proteins to allow optimal and efficient immune responses. Discovery of otulipenia/ORAS, HA20, and LUBAC-associated diseases has reiterated the importance of ubiquitination in regulation of immune signaling and revealed cell-specific functions of these proteins.

Despite similar function of OTULIN and A20 in restricting immune responses, the DUB activity of A20 appears to be less critical than the one of OTULIN (22). This may explain a milder inflammatory phenotype in HA20 than in patients with otulipenia. In addition, patients with otulipenia have more profound protein deficiency (less than 50%) than the patients with HA20 who retain one functional allele of the gene (50% protein deficiency).

Given the importance of the ubiquitination in cellular physiology, the UPS system has elicited a significant interest for drug development. The list of human diseases related to abnormalities in UPS has been steadily increasing and includes neurodegenerative diseases, cancer, and immune diseases. Ub-mediated protein degradation is critical for homeostasis in aging neuronal cells (67). Deficiency of A20 has been linked to lymphomas, and its most aggressive subtype is associated with constitutive activation of NF-kB. Reconstitution of A20 in mutant cell lines induced apoptosis and suppressed tumor growth (47). In immune diseases, A20 and OTULIN might be new therapeutic targets for development of immunomodulatory drugs that can potentially increase or stabilize their expression.

A key challenge for finding effective drugs will be in developing cell-based therapies. The ubiquitination process is regulated at multiple levels: generation, recognition, and removal. Targeting more components of the Ub–proteasome pathway may provide new opportunities for therapeutic exploitation and drug discovery (68, 69).

## Author Contributions

IA reviewed the literature and wrote the manuscript. QZ reviewed the literature to make figures and tables and helped with writing the manuscript.

## Conflict of Interest Statement

The authors declare that the research was conducted in the absence of any commercial or financial relationships that could be construed as a potential conflict of interest. The reviewer, JY, and handling Editor declared their shared affiliation, and the handling Editor states that the process nevertheless met the standards of a fair and objective review.
